# Supplementary figures and images for: Genome-Wide Identification and Analysis of the GRAS Transcription Factor Gene Family in Theobroma cacao
Source: Genes (Basel). 2022 Dec 24;14(1):57. doi: 10.3390/genes14010057 (PMC9858872; doi:10.3390/genes14010057)

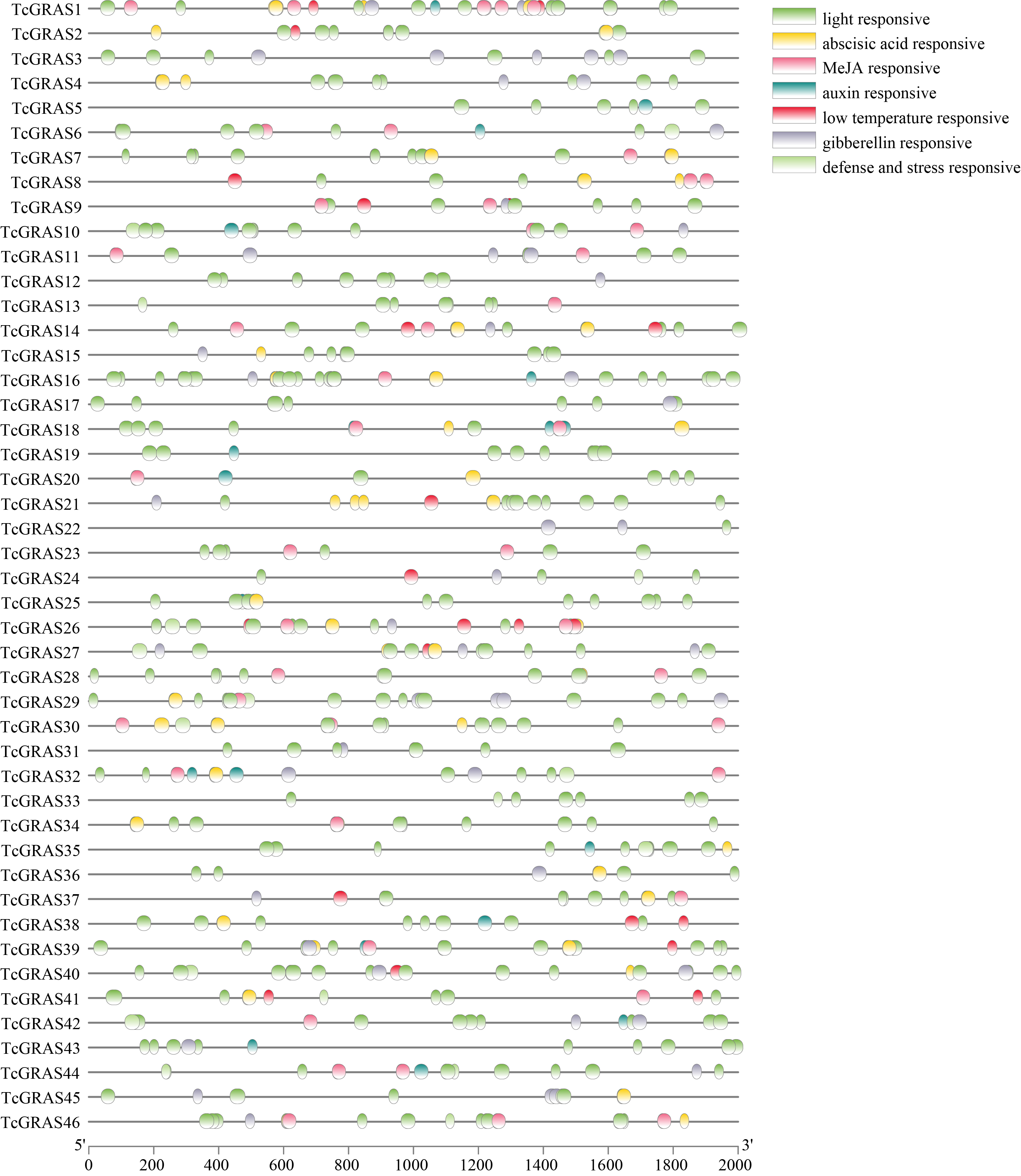

Supplement: Supplementary file 1 [file genes-14-00057-s001.zip › Supplementary File S3-The predicted cis-regulatory elements in promoters of TcGRAS genes.png]
